# Prognostic value of sarcopenia in patients with colorectal liver metastases undergoing hepatic resection

**DOI:** 10.1038/s41598-020-63644-x

**Published:** 2020-04-15

**Authors:** Yueh-Wei Liu, Chien-Chang Lu, Ching-Di Chang, Ko-Chao Lee, Hong Hwa Chen, Wen Shuo Yeh, Wang-Hseng Hu, Kai-Lung Tsai, Cheng-Hsi Yeh, Sin-Yong Wee, Shin-Min Yin, Chih-Chi Wang, Chao-Hung Hung

**Affiliations:** 1 Liver Transplant Center, Department of Surgery, Kaohsiung Chang Gung Memorial Hospital, and Chang Gung University College of Medicine, Kaohsiung, Taiwan; 2 Department of Colorectal Surgery, Kaohsiung Chang Gung Memorial Hospital and Chang Gung University College of Medicine, Kaohsiung, Taiwan; 3 Department of Radiology, Kaohsiung Chang Gung Memorial Hospital and Chang Gung University College of Medicine, Kaohsiung, Taiwan; 4 Division of Hepatogastroenterology, Department of Internal Medicine, Kaohsiung Chang Gung Memorial Hospital and Chang Gung University College of Medicine, Kaohsiung, Taiwan; 5 Division of Hepatogastroenterology, Department of Internal Medicine, Chiayi Chang Gung Memorial Hospital, Chiayi, Taiwan

**Keywords:** Gastroenterology, Liver cancer

## Abstract

The prognostic significance of sarcopenia has been widely studied in different cancer patients. This study aimed to analyze the influence of sarcopenia on long-term survival in patients with colorectal liver metastasis (CRLM) undergoing hepatic resection. A retrospective analysis of 182 patients undergoing hepatic resection for CRLM was performed. Sarcopenia was determinedusing the Hounsfield unit average calculation (HUAC), a measure of muscle quality-muscledensity at preoperative abdominal *computed tomography* scans. Sarcopenia was defined as an HUAC score of less than 22 HU calculated using receiver operating characteristic analysis. The prognostic relevance of clinical variables and overall survival (OS) and recurrence-free survival (RFS) was evaluated. Patients with sarcopenia were older (*p* < 0.001) and had higher prevalence of diabetics (*p* = 0.004), higher body mass index (BMI) (*p* < 0.001) and neutrophil-to-lymphocyte ratio (*p* = 0.026) compared to those without. Sarcopenia was not significantly associated with OS and RFS. Multivariate Cox’s regression analysis showed that multinodularity (>3) (hazard ratio (HR) 2.736; 95% confidence interval (CI), 1.631–4.589; *p* < 0.001), high CEA level (≥20 ng/ml) (HR 1.793; 95% CI, 1.092–2.945; *p* = 0.021) and blood loss (≥300 cc) (HR1.793; 95% CI, 1.084–2.964; *p* = 0.023) were independent factors associated with OS. In subgroup analyses, sarcopenia was a significant factor of poor OS in the patients with multinodularity by univariate (*p* = 0.002) and multivariate analyses(HR 3.571; 95% CI, 1.508–8.403; *p* = 0.004). Multinodularity (>3) (HR 1.750; 95% CI, 1.066–2.872; *p* = 0.027), high aspartate aminotransferase level (HR 1.024; 95% CI, 1.003–1.046; *p* = 0.025) and male gender (HR 1.688; 95% CI, 1.036–2.748; *p* = 0.035) were independent factors of RFS. In conclusion, despite no significance in whole cohort, sarcopenia was predictive of worse OS in patients with multiple CRLM after partial hepatectomy.

## Introduction

Colorectal canceris one of the leading malignanciesand the fifth most frequent cause of cancer-related deathworldwide^1,2^. Approximately 20–25% of patients have synchronous liver metastaseswhile being diagnosed, and a further 35 to 45% of patients will develop metachronoushepatic metastases following the removal ofprimary tumor^3^. The mean survival for patients with untreated colorectal liver metastasis (CRLM) has been found to range from 6–13 months^4,5^. Thus, the management of CRLM remains clinically challenging.

Hepatic resection is the mainstay of treatmentand potentially curative therapy for CRLM,with reported 5-year survival of 30–50%^4–6^. However, the recurrence rate has been reported to be high(60–80%)and only 16% of these patients remain disease free for 10 years after hepatectomy^7^. Some studies have therefore tried to find probable prognostic predictors affecting CRLM receiving resection, including elder age, number and size of hepatic lesions, the primary lymph node status and preoperative anemia^8,9^. This issue should be further clarified with the advances of new chemotherapeutic regimensand target therapy as well as the operative technique.

Sarcopenia is a syndrome characterized by loss of muscle mass, function and strength that is quantifiable using cross sectional imaging by measurement of psoas area and the muscle’s density^10,11^. Recently, there is increasing evidence that sarcopeniais an important prognostic factor of frailty, mortality, and worse surgical outcomes^12^. Sarcopenia has been reported to affect not only operative complications^13^, but also cancer-specific outcomes following hepatic resection^14,15^, colectomy^16^, and pancreatic resection^17^. Until now, the role of sarcopenia in the long-term survival of patients with CRLMafter partial hepatectomy remains limited^15,18^. Thus, we conducted this study to clarify this issue.

## Materials and methods

### Study population

Between July 2008 and July 2018, 193 consecutive patients who underwent curative intent surgery for CRLM were identified from a single tertiary center. Of them, 11 patients without available abdominal computed tomography (CT) images within 30 days of surgerywere excluded. Preoperative workup included triple phase-contrast enhanced CT scan, in which liver volume assessment was performed when indicated. Magnetic resonance imaging (MRI) and/or positron emission tomography (PET) scan were arranged to confirm doubtful cases. These cases were discussed in a multidisciplinary meeting to decide combined or delayed surgery with or without pre-operativechemotherapy. Written informed consent was obtained from all participants. This study was approved by the Research Ethics Committee of Chang Gung Memorial Hospital (IRB No. 201900541B0) and was conducted in accordance with the principles of Declaration of Helsinki and the International Conference on Harmonization for Good Clinical Practice.

### Image analysis

Preoperative abdominal CT scans were performed on a GE Discovery CT750 HD lightspeed scanner. Evaluation for sarcopenia was performed using CT measures ofmuscle quality-muscledensity (measured in Hounsfield units (HU))as previously described^19–21^. All measurements and segmentations were done at the level of the inferior endplate of L4 on axial CT images. To measure muscle density, the paraspinal muscles were outlined using a freehand region-of-interest (ROI) tool on a General Electric Picture Archiving and Communicating System (S1000). The total cross-sectional area of bilateral paraspinal, psoas, and abdominal wall muscles at L4 were evaluated on OsiriX imaging software v. 8.0.2 (Pixmeo, Geneva, Switzerland) with the lean muscle threshold set at −29 to 150 HU^19–21^. The final Hounsfield unit average calculation (HUAC)was the average ofleft HU and right HU to determine the presence or absence of sarcopenia in the study population.

### Follow-up

The regimens of chemotherapy included fluorouracil and leucovorin, combined with irinotecan and/or oxaliplantin, which was currently the standard treatment for CRLM. Follow-up consisted of out patient visits with serum carcinoembryonic antigen (CEA) levels and imagesevery 3 to 6 months after surgery. Overall survival (OS) was defined as the time interval from the date of hepatic resectionto death from any cause or the last follow-up date. Recurrence-free survival (RFS) was defined as the period after hepatic resection to the date when recurrent tumors were diagnosed.

### Statistical analysis

Continuous data were presented as mean ± standard deviation and compared by using Student’s *t* test. Categorical variables were expressed as number (percentage) and analyzed by using the χ^2^ or Fisher’s exact test depending on the size of the sample. A receiver-operating characteristic (ROC) curve was used to determine the best cutoff of HUAC scorebased on the Youden index. Kaplan–Meier curves were generated for OS and RFS and the differences of survival rates between groups were compared using the log-rank test. The Cox proportional hazards model was employed for univariate and multivariate analyses. The analysis software used was SPSS for Windows version 18 (SPSS Inc., Chicago, IL, USA). All statistical tests were two-sided and differences were considered significant with a *p* < 0.05.

## Results

### Patient characteristics

Among the study population, the mean age was 59.5 ± 12.1 years with a range 21–85 years, and 106 of them were male. Sixty-twopatients had solitary tumor whereas 57 had 2–3 tumors, and 63 had more than 3 tumors. Neoadjuvant chemotherapy was given in 35 of 182 patients (19.2%), and 166(91.2%) patients received adjuvant chemotherapy after hepatic resection (Table 1).

**Table 1:** Baseline characteristics of the study cohort.

Age (years)	59.5 ± 12.1
Gender Male/Female	106/76
Body mass index (kg/m^2^)	24.3 ± 3.6
Type 2 DM (%)	40 (22%)
Synchronous(%)	119 (65%)
**Chemotherapy**	
Neoadjuvant/adjuvant/both	35/166/28
Primary tumor site*	
C/A/T/D/S/RS/R	5/14/9/18/69/12/55
Tumor stage T1/T2/T3/T4	3/22/73/84
Nodal status N0/N1/N2	54/83/45
Main tumor (cm)	3.9 ± 2.5
Tumor number1/2,3 />3	62/57/63
CEA (ng/ml)	178 ± 880
AST (U/L)	29 ± 13
ALT (U/L)	27 ± 22
Albumin (g/dl)	4.1 ± 0.4
Platelet (10^3^/μL)	257 ± 98
NLR	2.9 ± 2.3

^*^C/A/T/D/S/RS/R: cecum/ascending colon/transverse colon/descending colon/sigmoid colon/recto-sigmoid colon/rectum.Abbreviation: DM, diabetes mellitus; CEA, c*arcinoembryonic antigen*; aspartate aminotransferase; ALT, alanine aminotransferase; NLR, neutrophil-to-lymphocyte ratio.

ROC analysis for HUAC score in the survival status at the 5-year follow-up identified an optimal cutoffat HUAC of 22 HU. At this cutoff, 48 (26.4%) patients were considered sarcopenic. Patients with sarcopenia were older (*p* < 0.001) and had higher prevalence of diabetics (*p* = 0.004), higher body mass index (BMI) (*p* < 0.001) and neutrophil-to-lymphocyte ratio (*p* = 0.026) compared to those without sarcopenia (Table 2). Themajor complications were equally common in sarcopenic and non-sarcopenic patients.

**Table 2:** Comparison of demographic and clinical characteristics of patients with and without sarcopenia.

	With Sarcopenia	Without Sarcopenia	*p*-value
Case number	48	134	
Age (years)	66.6 ± 10.2	57.0 ± 11.7	<0.001
Male (%)	24(50%)	82(61%)	0.232
Body mass index (kg/m^2^)	25.9 ± 4.1	23.8 ± 3.2	<0.001
Type 2 DM (%)	18(38%)	22(16%)	0.004
Synchronous (%)	27(56%)	92(69%)	0.157
Neoadjuvant chemotherapy	10 (21%)	25 (19%)	0.831
Adjuvant chemotherapy	43 (90%)	123 (92%)	0.767
Right-sided primary tumor (%)	6(13%)	22(16%)	0.644
Tumor stage T3, 4 (%)	41(85%)	116(87%)	0.811
Nodal status N2 (%)	10 (21%)	35(26%)	0.560
Main tumor (cm)	4.1 ± 2.3	3.8 ± 2.5	0.453
Tumor number >3 (%)	14(29%)	49(37%)	0.382
CEA (ng/ml)	356 ± 1412	114 ± 571	0.102
AST (U/L)	28 ± 14	29 ± 13	0.575
ALT (U/L)	22 ± 12	29 ± 23	0.153
Albumin (g/dl)	3.9 ± 0.8	4.1 ± 0.5	0.072
Platelet (10^3^/μL)	305 ± 124	295 ± 102	0.601
NLR	3.5 ± 3.4	2.7 ± 1.7	0.026

Data are expressed as mean±standard deviation or number (percentage).

Abbreviation: DM, diabetes mellitus; CEA, c*arcinoembryonic antigen*; aspartate aminotransferase; ALT, alanine aminotransferase; NLR, neutrophil-to-lymphocyte ratio.

### Factors associated with OS

**Figure 1:**
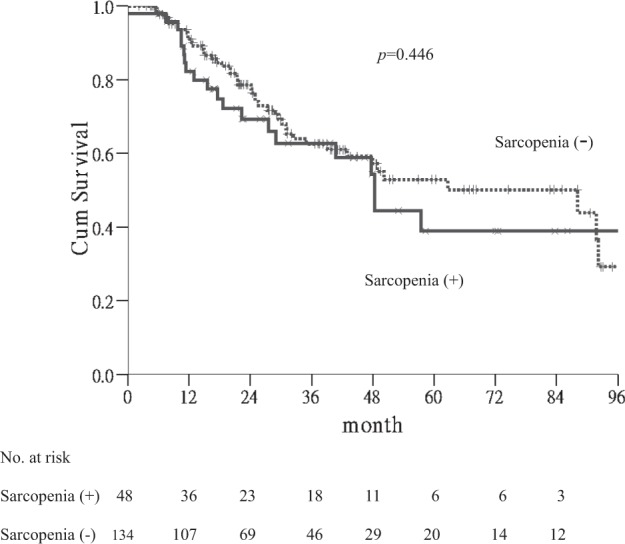
Kaplan-Meier curves for overall survival. Significance was not found between patients with and without sarcopenia(*p* = 0.446).

The mean follow-up was 32.5 ± 25.5 months (range 2–121). The 1-, 3-, and 5-year OS rates of patients were 89%, 63%, and 49%, respectively. Sarcopenia was not a prognostic factor for OS, as the mean OS durations for patients with sarcopenia was 32.3 ± 25.0 months vs. 33.0 ± 27.1 months for those without sarcopenia (Fig. 1).

Univariate analyses showed significant associations of unfavourable OS were multinodularity (>3) (*p* < 0.001), main tumor size (≥3 cm) (*p* = 0.023), blood loss (≥300 cc) (*p* = 0.003), CEA level (≥20 ng/ml) (*p* = 0.046) and platelet count (*p* = 0.016). By multivariate Cox proportional hazards model of factors with p < 0.2 in univariate analyses, multinodularity (hazard ratio (HR) 2.736; 95% confidence interval (CI), 1.631–4.589; *p* < 0.001), high CEA level (≥20 ng/ml) (HR 1.793; 95% CI, 1.092–2.945; *p* = 0.021) and blood loss (≥300 cc) (HR 1.793; 95% CI, 1.084–2.964; *p* = 0.023) were independent factors for OS (Table 3).

**Table 3:** Univariate and stepwise multivariate analyses of factors associated with overall survival.

	Comparison	Univariate analyses		Stepwise multivariate analyses	
		HR (95% CI)	*p*-value	HR (95% CI)	*p*-value
Age (years)	≥60 vs. <60	0.785 (0.483–1.276)	0.328		
Gender	Male vs. Female	1.132 (0.694–1.849)	0.619		
BMI(kg/m^2^)	≥25 vs. <25	0.683 (0.410–1.135)	0.141		
DM	Yes vs. No	1.365 (0.785–2.374)	0.270		
Sarcopenia	Yes vs. No	1.226 (0.725–2.073)	0.447		
Synchronous	Yes vs. No	0.911 (0.545–1.522)	0.722		
Neoadjuvant chemotherapy	Yes vs. No	1.116 (0.596–2.092)	0.731		
Adjuvant chemotherapy	Yes vs. No	1.303 (0.522–3.250)	0.571		
Blood loss (cc)	≥300 vs. <300	2.116 (1.293–3.461)	0.003	1.793(1.084–2.964)	0.023
CEA (ng/ml)	≥20 vs. <20	1.639 (1.008–2.663)	0.046	1.793 (1.092–2.945)	0.021
Primary tumor site	right vs. left	1.530 (0.775–3.021)	0.220		
Tumor stage	T3,4 vs. 2	1.305 (0.622–2.735)	0.481		
Nodal status	N2 vs. N0, 1	1.639 (0.981–2.739)	0.059		
Main tumor (cm)	>3 vs. ≤3	1.783 (1.085–2.930)	0.023		
Tumor number	>3 vs. ≤3	2.733 (1.675–4.459)	<0.001	2.736 (1.631–4.589)	<0.001
AST (U/L)	per 1 U/L increase	1.016 (0.999–1.032)	0.068		
ALT (U/L)	per 1 U/L increase	1.007 (0.992–1.022)	0.361		
Albumin (g/dl)	per 1 g/dl increase	0.744 (0.533–1.038)	0.082		
Platelet (10^3^/μL)	per 10^3^/μL increase	1.003 (1.001–1.005)	0.016		
NLR	> 3 vs. ≤3	1.055 (0.608–1.830)	0.849		

Abbreviation: HR, hazard ratio; CI, confidence interval; DM, diabetes mellitus; CEA, carcinoembryonic antigen;

aspartate aminotransferase; ALT, alanine aminotransferase; NLR, neutrophil-to-lymphocyte ratio.

**Figure 2:**
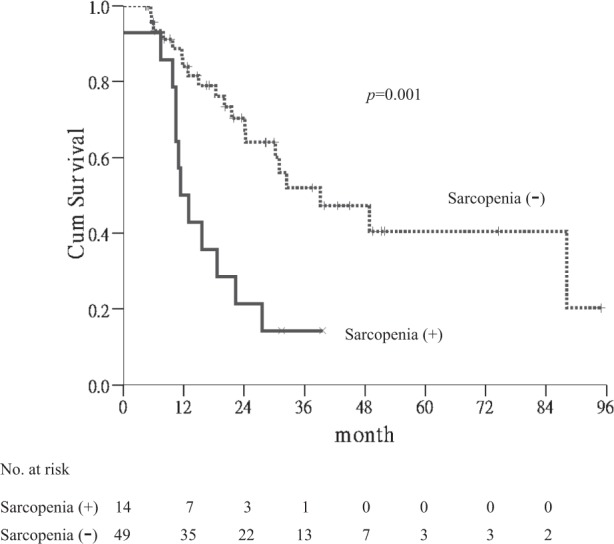
Kaplan-Meier curves for overall survival in subgroup patients. Sarcopenia was a significant factor of poor OS in the patients withmultinodularity (*p* = 0.001).

In subgroup analyses, sarcopenia was a significant factor of poor OS in the patients with multinodularity (*p* = 0.001) (Fig. 2). In contrast, there were no associations of sarcopenia with OS in other subgroup analyses, such as age, gender, diabetes, tumor size, etc. Table 4 shows thefactors associated with OS in subgroup patients with multiple CRLM. Sarcopenia was the significant factor associated with poor OS in the patients with multiple CRLM by univariate (*p* = 0.002) and multivariate analyses(HR 3.571; 95% CI, 1.508–8.403; *p* = 0.004).

**Table 4:** Factors associated with overall survival in subgroup patients with multiple CRLM.

	Comparison	Univariate analyses		Stepwise multivariate analyses	
		HR (95% CI)	*p*-value	HR (95% CI)	*p*-value
Age (years)	≥60 vs. <60	0.592 (0.291–1.202)	0.147		
Gender	Male vs. Female	1.151 (0.563–2.352)	0.701		
BMI (kg/m^2^)	≥25 vs. <25	1.373 (0.672–2.806)	0.384		
DM	Yes vs. No	1.078 (0.515–2.258)	0.842		
Sarcopenia	Yes vs. No	3.322 (1.585–6.993)	0.002	3.571 (1.508–8.403)	0.004
Synchronous	Yes vs. No	1.866 (0.755–4.610)	0.177		
Neoadjuvant chemotherapy	Yes vs. No	1.226 (0.589–2.551)	0.585		
Adjuvant chemotherapy	Yes vs. No	0.298 (0.088–1.008)	0.051		
Blood loss (cc)	≥300 vs. <300	1.940 (0.956–3.936)	0.067		
CEA (ng/ml)	≥ 10 vs. <10	1.627 (0.808–3.273)	0.173		
Primary tumor site	right vs. left	1.699 (0.645–4.475)	0.283		
Tumor stage	T3,4 vs. 2,1	1.147 (0.348–3.787)	0.821		
Nodal status	N2 vs. N0, 1	1.124 (0.536–2.353)	0.757		
Main tumor (cm)	>3 vs. ≤3	1.822 (0.865–3.836)	0.114		
AST (U/L)	per 1 U/L increase	1.000 (0.979–1.021)	0.966		
ALT (U/L)	per 1 U/L increase	0.998 (0.982–1.014)	0.776		
Albumin (g/dl)	per 1 g/dl increase	0.356 (0.115–1.098)	0.072		
Platelet (10^3^/μL)	per 10^3^/μL increase	1.002 (0.997–1.008)	0.353		
NLR	> 3 vs. ≤3	1.234 (0.563–2.703)	0.599		

Abbreviation: CRLM, colorectal liver metastasis; HR, hazard ratio; CI, confidence interval; DM, diabetes mellitus; CEA, carcinoembryonic antigen; aspartate aminotransferase; ALT, alanine aminotransferase; NLR, neutrophil-to-lymphocyte ratio.

### Factors associated with RFS

**Figure 3:**
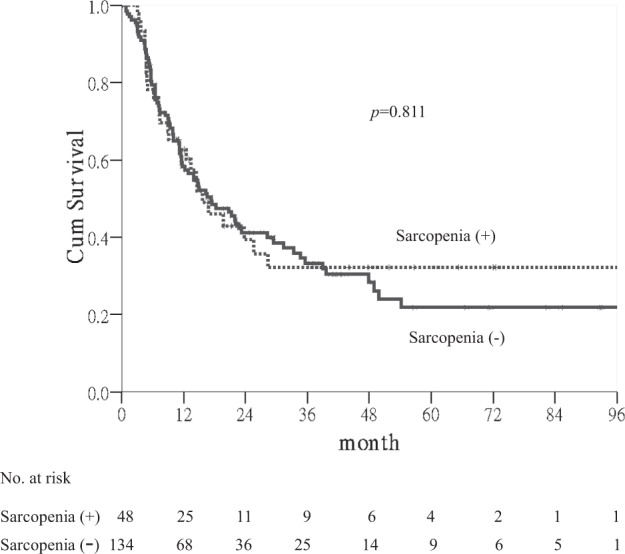
Kaplan-Meier curves for recurrence-free survival. Significance was not found between patients with and without sarcopenia(p = 0.811).

The 1-, 3-, and 5-year RFS rates of patients were 59%, 33%, and 25%, respectively. Sarcopenia was not a significant factor for RFS (Fig. 3). As shown in Table 5, multinodularity (*p* < 0.001), main tumor size (≥3 cm) (*p* = 0.017), male gender (*p* = 0.035) and higher pretreatment serum levels of aspartate aminotransferase (AST) (*p* = 0.004) and alanine aminotransferase (ALT) (*p* = 0.008) were associated with shorter RFS. Multivariate Cox’s regression analyses showed that multinodularity (>3) (HR 1.750; 95% CI, 1.066–2.872; *p* = 0.027), high AST level (HR 1.024; 95% CI, 1.003–1.046; *p* = 0.025) and male gender (HR 1.688; 95% CI, 1.036–2.748; *p* = 0.035) were independent factors (Table 5).

**Table 5:** Univariate and stepwise multivariate analyses of factors associated with recurrence -free survival.

	Comparison	Univariate analyses		Stepwise multivariate analyses	
		HR (95% CI)	*p*-value	HR (95% CI)	*p*-value
Age (years)	≥60 vs. <60	0.897 (0.617–1.305)	0.571		
Gender	Male vs. Female	1.519 (1.030–2.239)	0.035	1.688 (1.036–2.748)	0.035
BMI (kg/m2)	≥25 vs. <25	0.937 (0.637–1.377)	0.739		
DM	Yes vs. No	1.073 (0.681–1.689)	0.762		
Sarcopenia	Yes vs. No	1.054 (0.683–1.628)	0.811		
Synchronous	Yes vs. No	1.129 (0.760–1.678)	0.548		
Neoadjuvant chemotherapy	Yes vs. No	0.854 (0.521–1.401)	0.533		
Adjuvant chemotherapy	Yes vs. No	1.814 (0.839–3.919)	0.130		
Blood loss (cc)	≥300 vs. <300	1.451 (0.987–2.134)	0.058		
CEA (ng/ml)	≥20 vs. <20	1.374 (0.931–2.029)	0.110		
Primary tumor site	right vs. left	0.704 (0.394–1.260)	0.238		
Tumor stage	T3,4 vs. 2,1	1.538 (0.844–2.803)	0.160		
Nodal status	N2 vs. N0, 1	1.259 (0.827–1.918)	0.283		
Main tumor (cm)	>3 vs. ≤3	1.588 (1.086–2.322)	0.017		
Tumor number	>3 vs. ≤3	2.222 (1.505–3.281)	<0.001	1.750 (1.066–2.872)	0.027
AST (U/L)	per 1 U/L increase	1.021 (1.006–1.035)	0.004	1.024 (1.003–1.046)	0.025
ALT (U/L)	per 1 U/L increase	1.016 (1.004–1.027)	0.008		
Albumin (g/dl)	per 1 g/dl increase	1.025 (0.738–1.423)	0.883		
Platelet (10^3^/μL)	per 10^3^/μL increase	0.999 (0.998–1.001)	0.594		
NLR	>3 vs. ≤3	0.984 (0.651–1.488)	0.939		

Abbreviation: HR, hazard ratio; CI, confidence interval; DM, diabetes mellitus; CEA, carcinoembryonic antigen.

aspartate aminotransferase; ALT, alanine aminotransferase; NLR, neutrophil-to-lymphocyte ratio.

## Discussion

This present study showed that preoperative sarcopenia was not associated with long-term survival in a homogeneous population of CRLM undergoing hepatic resection. Sarcopeniawas not a significant risk factor of OS and RFS in our study population. However, our study provided the first evidence that sarcopenia predicted worse OS in the subgroup patients with multinodularity (>3).

A number of clinicopathological factors have been constantly reported as having prognostic value following hepatectomy of CRLM^8,9,22–24^. In this study, we demonstrated that multinodularity (>3), high CEA level (≥20 ng/ml) and blood loss (>300 cc) were independent factors associated with poorer OS. Our data were compatible with those reported in most studies^8,9,22–24^, showing that the number of liver metastaseswas the most important negative predictor not only for OS but also for RFS. In contrast, the location of primary tumoremerging as an important prognostic factor was not significant in our study^25^. This could be attributed to the small case number of right sided cancer in this series.

Recently, the use of sarcopenia to predict outcomes in cancer patients has attracted more attention, including those with CRLM undergoing hepatic resection. Previous studies demonstrated that sarcopenia increased risk of post-operative morbidity and longer hospital stay as well as readmission ratesafter partial liver resection for CRLM^15,18^. On the contrary, sarcopeniadid not seemto impact long-term outcomes in their patients. Our data, in line with their results, showed that sarcopenia was not a significant risk factor of OS and RFS. Moreover, considering the greater impact of other stronger risk factors such as tumor number, we therefore analyzed the effect of sarcopenia on the subgroups of our patients. Interestingly, we found that sarcopenia was significantly predictive of worse OS in the patients with multiple CRLM. Based on our findings, we recommended that patients with multiple (>3) CRLM and combined sarcopenia, hepatic resection might be considered cautiously due to limited survival. However, further studies with longer follow-up periods should be necessary to confirm our observation.

In our study population, sarcopenia was associated with advanced age, diabeticsand obesity. Although these results were discrepant with other findings^15,26^, our data were consistent with a recent report showing that age and obesity were found to be independently associated with sarcopenia in patients undergoing liver transplant evaluation^27^. Previous studies have reported that patients with sarcopenic obesity had worse survival in hepatocellular carcinoma receiving hepatectomy or after living donor liver transplantation^28–31^. While our data in accordance with a recent study showed that sarcopenicobesity was not a prognostic factor in patientsundergoingliver resection for CRLM^32^.

This present study is limited based on its retrospective nature and thus there may have been selection bias. Moreover, we believed that the bias was smallbecause patientswere followed regularly with clinical and laboratory assessment using CEA and imaging studies every 3 to 6 months. Secondly, we defined obesity as BMI≧25 kg/m^2^ by clinical diagnosis, whereas other studies assessed the visceral adipose tissueusing CT evaluation^18,29^. However, a previous study has showna close positive correlation of BMI with visceral adipose tissue, andobesity is adequately specified as a BMI≧25 kg/m^2^ in Asian populations^33^. Third, the recent novel mutational molecular markers, such as microsatellite instability, BRAF, and KRAS/NRAS and combination mutations which conferred poorer outcomeswere not available in our study^24^. It willbe interesting to determine the association of molecular markerswith long-term prognosisin patients with CRLM after partial hepatectomy in further studies.

In conclusion, in spite of no significance in long-term outcomes in whole cohort, sarcopenia is associated with an increased survival risk of patients with multiple CRLM undergoing hepatic resection. Assessment of preoperative sarcopenia provides an easy tool for selection of CRLM patients for liver resection. Further large-scale and multicenter studies are stillneeded to clarify these issues.
